# Sulforaphane Alleviates α-Amanitin-Induced Liver Injury by Modulating Pink1-Parkin Pathway

**DOI:** 10.3390/nu18193282

**Published:** 2026-10-06

**Authors:** Jian Sun, Yangyang Li, Zehui Li, Zhijie Liu, Zhengwang Yi, Peng Wu, Juyu Wang, Weijie Qu, Bundit Tengjaroenkul, Yi Wu, Xiaolong Gu

**Affiliations:** 1 Department of Clinical Veterinary Medicine, College of Veterinary Medicine, Yunnan Agricultural University, Jin Hei Road No. 95, Panlong District, Kunming 650051, China; 2 Yunnan Plateau Characteristic Agricultural Industry Research Institute, Yunnan Agricultural University, Kunming 650201, China; 3 Faculty of Veterinary Medicine, Khon Kaen University, Khon Kaen 40002, Thailand

**Keywords:** Sulforaphane (SFN), α-amanitin (α-AMA), Pink1-Parkin, oxidative stress, apoptosis

## Abstract

Background: α-Amanitin (α-AMA) is the primary lethal toxin found in poisonous mushrooms of the genus Amanita; accidental ingestion can lead to severe liver injury, and there is currently no specific antidote. Due to its significant anti-inflammatory and antioxidant activity, Sulforaphane (SFN) has attracted increasing attention for its role in preventing liver injury. However, it remains unclear whether SFN can alleviate α-AMA-induced liver injury. This study aimed to elucidate the protective effects of SFN against α-AMA-induced liver injury and its potential molecular mechanisms. Methods and Results: The results showed that SFN significantly reduced serum ALT and AST levels in α-AMA-intoxicated mice (*p* < 0.01), alleviated pathological damage such as hepatic edema, lobular rupture, and inflammatory infiltration, reduced apoptosis, lowered MDA levels, and simultaneously increased CAT activity (*p* < 0.01). In AML-12 cells, SFN effectively reversed α-AMA-induced cellular damage (including reduced cell viability and decreased mitochondrial membrane potential). This study further found that SFN significantly upregulated the expression of Pink1 and Parkin. Furthermore, the overexpression of Pink1 significantly alleviated α-AMA-induced oxidative stress, mitochondrial damage, and apoptosis. Conclusions: The above results indicate that SFN may alleviate α-AMA-induced liver damage by promoting the expression of Pink1 and Parkin, improving mitochondrial dysfunction, and inhibiting apoptosis.

## 1. Introduction

Sulforaphane (SFN) is a naturally occurring isothiocyanate compound that is found in cruciferous vegetables, such as broccoli and Chinese kale, in particular in the seeds and buds of the latter. In recent years, the research of SFN in the field of liver injury protection is increasing. A number of studies have shown that SFN can reduce different types of liver injury through a variety of mechanisms [1]. In the liver injury model induced by exhaustive exercise, SFN intervention can significantly reduce serum lactate dehydrogenase, creatine kinase and transaminase levels and reduce oxidative stress and inflammatory response [2]. In addition, SFN has been demonstrated to exert a protective effect on LPS-induced liver injury by activating AMPK to enhance antioxidant and anti-apoptotic capabilities [3]. In the non-alcoholic fatty liver disease (NAFLD) model, SFN can reduce fat accumulation in hepatocytes by regulating the FXR-LXRα signaling pathway [4]. It can also improve intestinal barrier function and reduce LPS translocation through the gut-liver axis [5,6,7]. The preceding studies have demonstrated, as a naturally occurring dietary ingredient, good protective effects in a variety of liver injury models and has the potential to be developed as a functional food.

α-Amanitin (α-AMA) is the primary lethal toxin found in poisonous mushrooms of the genus Amanita. The ingestion of mushrooms containing α-AMA has been demonstrated to result in severe liver damage and even acute liver failure. Studies have shown that α-AMA blocks protein synthesis mainly by inhibiting the activity of RNA polymerase II, while inducing oxidative stress, mitochondrial damage and apoptosis [8]. It has been demonstrated that α-AMA has the capacity to induce an inflammatory response, which is a process that is initiated by the activation of the NLRP3 signalling pathway [9], and can also aggravate liver damage by inhibiting PPAR-γ and activating the NLRP3 inflammasome [10]. It is worth noting that α-AMA can also activate oxidative stress and mitophagy by inhibiting PRDX6 expression, thereby aggravating liver damage [11]. Currently, there is no specific clinical antidote for α-AMA poisoning, and early detection and plasma exchange are the main treatments [12]. In recent years, there has been a growing interest in the application of natural products in the context of α-AMA detoxification. Research has demonstrated that resveratrol has the capacity to exert a protective effect on α-AMA-induced hepatotoxicity [13], and ganoderic acid A can reduce α-AMA-induced liver damage by regulating multiple metabolic pathways [14]. These findings suggest that natural active substances have potential application value in α-AMA detoxification. However, further research is required to elucidate the protective effect and mechanism of SFN on α-AMA-induced liver injury.

Pink1 (PTEN-induced putative kinase 1) is a serine-threonine protein kinase that is localised in the mitochondria. It has been demonstrated that this protein functions as a regulator of mitophagy, a process that involves the removal of damaged or dysfunctional mitochondria within cells. In addition to its role in mitophagy, this protein has been shown to play a pivotal part in maintaining the quality of mitochondria and preserving cellular homeostasis. A plethora of studies have demonstrated that Pink1-Parkin-mediated mitophagy plays a pivotal role in the protection of various liver injury models. In the condition known as NAFLD, the process of mitophagy, or the elimination of damaged or superfluous mitochondrial components, has been observed to be activated by the Pink1-Parkin pathway. This activation has been shown to result in a reduction in the accumulation of lipids within the liver, as well as a corresponding alleviation of mitochondrial damage [15,16]. In a high-fat diet-induced metabolism-related fatty liver disease model, myricetin activates mitophagy by stabilizing Pink1 and improves hepatic steatosis and inflammation [17]. In liver fibrosis research, SIRT3 regulates mitophagy by deacetylating Pink1 and NIPSNAP1, thereby alleviating fibrosis progression [18]. In addition, Pink1-Parkin-mediated mitophagy also plays a protective role in liver injury induced by aflatoxin B1, sodium fluoride and other poisons [19,20]. In α-AMA-induced liver injury, activation of mitophagy can significantly reduce liver cell damage, suggesting that the Pink1-Parkin pathway may be an important target for α-AMA detoxification [21]. However, it is unknown whether SFN alleviates α-AMA-induced liver injury by regulating Pink1 expression. Based on this issue, we plan to explore whether SFN has a protective effect on liver injury caused by α-AMA.

## 2. Materials and Methods

### 2.1. Reagents and Instruments

Sulforaphane (MedChemExpress, Monmouth Junction, NJ, USA), α-amanitin (APExBIO, Houston, TX, USA), DMEM/F-12 liquid culture medium (ZEHAO TRADE Co., Ltd., Kunming, China), fetal bovine serum (FBS) (ZETA LIFE, Menlo Park, CA, USA), penicillin-streptomycin double antibody, transmission electron microscope fixative (Servicebio, Wuhan, China), insulin-transferrin-selenium culture medium supplement (ITS), RIPA lysis buffer (strong), protease phosphatase inhibitor mixture (universal, 50×), mitochondrial membrane potential detection kit (TMRE), reactive oxygen species detection kit (Beyotime, Shanghai, China), cell-grade dexamethasone (BasalMedia, Shanghai, China), dimethyl sulfoxide (DMSO) (MACKLIN, Shanghai, China), BCA protein quantification kit, PAGE gel rapid preparation kit (Yase Biotechnology, Shanghai, China), PBS phosphate buffer, SDS-PAGE protein loading buffer (5×), SDS-PAGE Running buffer, SDS-PAGE Transfer buffer Powder, CCK-8 kit (LABGIC, Beijing, China), No protein blocking solution (2%) (Sangon Biotech, Shanghai, China), Protein Marker (UELandy, Suzhou, China), reactive oxygen species detection reagent Kit (Key GENBioTECH, Nanjing, China), anti-fluorescence attenuation mounting medium (containing DAPI), malondialdehyde (MDA) kit, catalase (CAT) kit, alanine aminotransferase (ALT) kit, aspartate aminotransferase (AST) kit (Solarbio, Beijing, China), Pink1 overexpression plasmid (GenePharma, Shanghai, China), Lip3000 transfection reagent (Thermo Fisher Scientific, Waltham, MA, USA). Antibody catalog numbers and dilution ratios used: β-actin (Proteintech, Rosemont, IL, USA, catalog number 66009-1-Ig, 1:1000), Pink1 (Proteintech, Rosemont, IL, USA, catalog number 23274-1-AP, 1:3000), Parkin (Cell Signaling Technology, MA, USA, catalog number 4211, 1:1000), Bax (Proteintech, Rosemont, IL, USA, catalog number 50599-2-Ig, 1:1000), Bcl-2 (Proteintech, Rosemont, IL, USA, catalog number 26593-1-AP, 1:1000) and Cleaved-Caspase-3 (Proteintech, Rosemont, IL, USA, catalog number 25128-1-AP, 1:1500).

### 2.2. Cell Culture

The AML-12 cells (purchased from Wuhan Pronosei Life Technology Co., Ltd., Wuhan, China) were cultivated in complete culture medium containing 10% FBS, 0.5% ITS and 40 ng/mL dexamethasone. The cells were then placed in an incubator set at 37 °C and 5% CO_2_.

### 2.3. Animals and Treatment

Male C57BL/6J mice, 6 to 8 weeks old, weighing approximately 20 ± 2 g, were purchased from Wuhan Beisai Model Biotechnology Company (Wuhan, China) (certificate number: SCXK (E) 2022-0029). They were kept in an environment of 25 ± 2 °C and 60 ± 5%, with free access to food and water. To minimise potential confounders, animals were randomly allocated to experimental groups using a computer-generated randomisation sequence (GraphPad Prism) with block randomisation. Randomisation was stratified by body weight. After 1 week of adaptive feeding, 40 mice were randomly divided into the control group, SFN (25 mg/kg) group, α-AMA group (0.3 mg/kg), and SFN (25 mg/kg) + α-AMA (0.3 mg/kg) group. Each group consisted of 10 mice, with each mouse serving as an independent experimental unit. Both SFN and α-AMA were first dissolved in DMSO and then diluted with saline to the target concentration; the control group received the corresponding vehicle. SFN (25 mg/kg) was administered via daily oral gavage on days 1–7, while α-AMA (0.3 mg/kg) was administered via oral gavage on days 4 and 7, respectively. Approximately 24 h after the final α-AMA exposure on day 7—that is, on day 8—blood and liver tissue were collected. All procedures were performed at the same time of day where possible. The investigator performing the measurements was blinded to group allocation. Non-invasive methods were used where possible, restraint was minimised, and handling was performed by familiar personnel. No procedures were performed without anesthesia or analgesia when such intervention was appropriate. The study was conducted in accordance with the Declaration of Helsinki, and approved by the Life Science Ethics Committee of Yunnan Agricultural University on 6 February 2025 (APYNAU202502026) and carried out according to the Guide for the Care Yunnan Agricultural University and Use of Laboratory Animals of the National Research Council.

### 2.4. Cell Viability Detection

The initial cell density of the AML-12 cells was established at 1 × 10^5^ cells per well, following seeding in a 96-well plate. When the confluence reached 80% to 90%, the cells were subjected to a 3 h pre-treatment with 30 μM SFN. Thereafter, the old solution was removed, the cells were washed with a PBS buffer, and then treated with 10 μM α-AMA working solution for 24 h. Subsequently, 90 μL of fresh serum-free cell culture medium and 10 μL of CCK-8 reagent were added to each well. The cells were then incubated in an incubator set to 37 °C, with 5% CO_2_ and in the dark for 1.5 h. Finally, the cells’ absorbency was detected.

### 2.5. HE Staining

Following fixation of liver tissue in 4% paraformaldehyde for a 24 h period, the tissue underwent a series of processing steps, including dehydration, clearing, and embedding in paraffin, with the objective of preparing 4-μm-thick sections. Following dewaxing and rehydration of the sections, hematoxylin and eosin (H&E) staining was performed using a ready-to-use H&E staining kit from Beijing Zhongshan Jinqiao Biotechnology Co., Ltd. (ZSGB-BIO, Beijing, China). The sections were then dehydrated in a gradient of ethanol, cleared with xylene, and mounted with neutral resin.

### 2.6. TUNEL Staining

After paraffin-embedded liver tissue sections fixed with polyformaldehyde were deparaffinized and rehydrated, they were placed in 10 mM citrate buffer (pH 6.0) and underwent antigen retrieval for 10 min at 121 °C and 0.10 MPa. Subsequently, the sections were incubated with Proteinase K at 37 °C for 20 min to permeabilize them, followed by a 60 min TUNEL reaction at 37 °C in the dark. The sections were treated with a 3% H_2_O_2_-methanol solution for 10 min at room temperature, protected from light, to block endogenous peroxidase; streptavidin-HRP working solution was then added, and the sections were incubated for 40 min in a humid chamber at 37 °C, followed by DAB staining. HRP catalyzes the formation of a brown precipitate from DAB; therefore, TUNEL-positive cell nuclei appear brown. TUNEL-positive cells were counted using ImageJ software (1.54u8), and consistent image acquisition and analysis standards were applied across all groups.

### 2.7. Western Blot

Liver tissue or AML-12 cells were collected in 1.5 mL EP tubes and lysed with RIPA buffer, which had been supplemented with protease and phosphatase inhibitors. Following homogenisation, the samples were subjected to a centrifugal process at a speed of 12,000 rpm for a duration of 15 min. Thereafter, the resultant upper layer, or ‘supernatant’, was collected for subsequent analysis. The protein concentration of the sample was determined using bicinchoninic acid (BCA). The proteins were then separated using 10% SDS-PAGE and transferred to a polyvinylidene fluoride (PVDF) membrane. The PVDF membrane was blocked with 5% skimmed milk, washed with TBST, and then incubated in primary antibody for 12 h at 4 °C. Following this step, the membrane was then subjected to an incubation with the relevant secondary antibody for a period of one hour at ambient temperature. Finally, chemiluminescence was used for development.

### 2.8. ROS and Mitochondrial Membrane Potential Detection

AML-12 cells were treated with 30 μM SFN. Following a 3 h period, the solution was removed and washed with PBS. Thereafter, the solution was treated with 10 μM α-AMA for a period of 24 h. The cells were loaded with 10 μM 2′,7′-dichlorofluorescein diacetate (DCFH-DA) by incubating with 1 mL of the probe working solution in an incubator maintained at 37 °C and containing 5% CO_2_ for a period of 25 min in the absence of light. Following the incubation period, the loading solution was removed and the cells were washed thoroughly in order to remove any unincorporated probe. Subsequently, the samples were observed under a fluorescence microscope.

Mitochondrial membrane potential (Δψm) was measured using the TMRE kit. Following treatment, AML-12 cells were washed with PBS, and 1 mL of TMRE staining working solution was added. The cells were then incubated in an incubator set to 37 °C, with 5% CO_2_ and in the dark for 35 min. The solution was removed, after which the Δψm was observed with a fluorescence microscope following washing.

### 2.9. Transmission Electron Microscope

Liver tissues were cut into 1–3 mm pieces, and AML-12 cells were collected into a 1.5 mL centrifuge tube. Both samples were placed on ice and fixed with 2.5% glutaraldehyde solution. After rinsing with PBS buffer, they were fixed in 1% osmic acid, then dehydrated with ethanol, infiltrated with a mixture of acetone and epoxy resin embedding agent, then embedded in paraffin and sectioned, and stained with uranyl acetate staining solution and lead citrate staining solution. Finally, the mitochondria were observed using transmission electron microscopy.

### 2.10. Data Analysis

The experimental data were then subjected to statistical analysis using GraphPad Prism 10 software. The results obtained are expressed as the mean ± standard error of the mean (SEM), based on the independent biological replicates that were actually included in each experiment. When the appropriate conditions were met, one-way ANOVA was used, and Tukey’s test was employed for pairwise comparisons between groups. No predefined inclusion or exclusion criteria were established. The statistical notation employed is as follows: The symbol “*” is used to indicate a comparison with the control group, while “#” indicates a comparison with the α-AMA group. Significant differences between the corresponding groups are denoted by “* *p* < 0.05”, “** *p* < 0.01”, “# *p* < 0.05” and “## *p* < 0.01”, respectively.

## 3. Results

### 3.1. SFN Alleviated α-AMA-Induced Liver Injury in Mice

**Figure 1:**
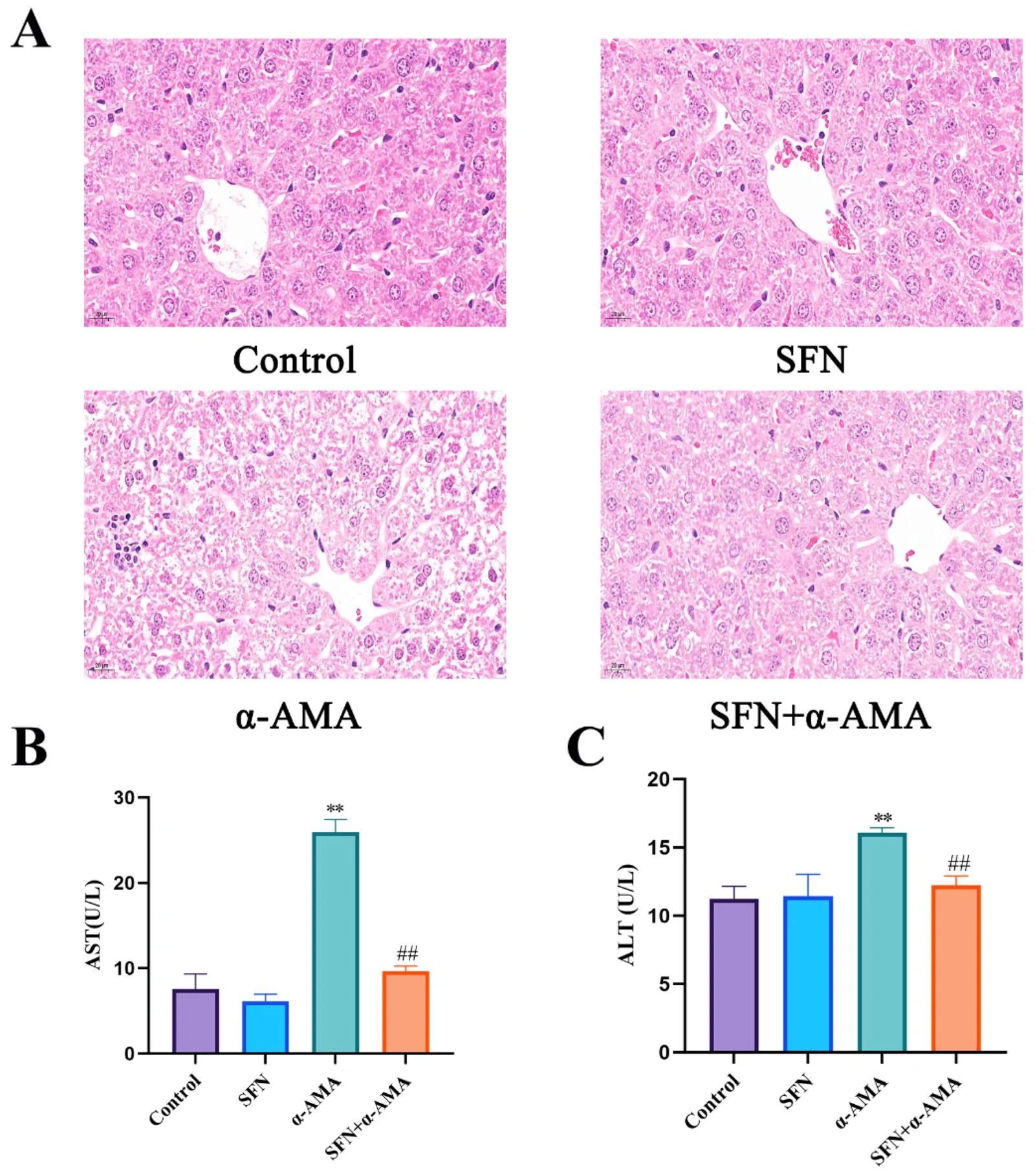
Protective effect of SFN on α-AMA-induced liver injury in mice. (**A**) HE staining of liver (scale bar: 50 μm). (**B**,**C**) Changes in AST and ALT levels. Note: *n* = 3; “*” indicates a significant difference compared with the control group (** *p* < 0.01); “#” indicates a significant difference compared with the α-AMA group (## *p* < 0.01).

In order to elucidate the effect of SFN on α-AMA-induced liver injury in mice, a control group, 25 mg/kg SFN group, 0.3 mg/kg α-AMA group and SFN (25 mg/kg) + α-AMA (0.3 mg/kg) group were set up. The results of HE staining were shown in Figure 1A. Compared with the control group, the α-AMA group had hepatocyte edema around the central vein of the liver, hepatic cord rupture, and more inflammatory cells. Compared with the α-AMA group, hepatocyte swelling was alleviated in the SFN+α-AMA group. The AST and ALT detection results were shown in Figure 1B,C. α-AMA caused a significant increase in ALT and AST levels in mice (*p* < 0.01). Treatment with SFN for one week significantly reduced AST and ALT levels (*p* < 0.01), indicating that SFN has a protective effect on α-AMA-induced liver injury in mice.

### 3.2. SFN Alleviated α-AMA-Induced Liver Oxidative Stress in Mice

**Figure 2:**
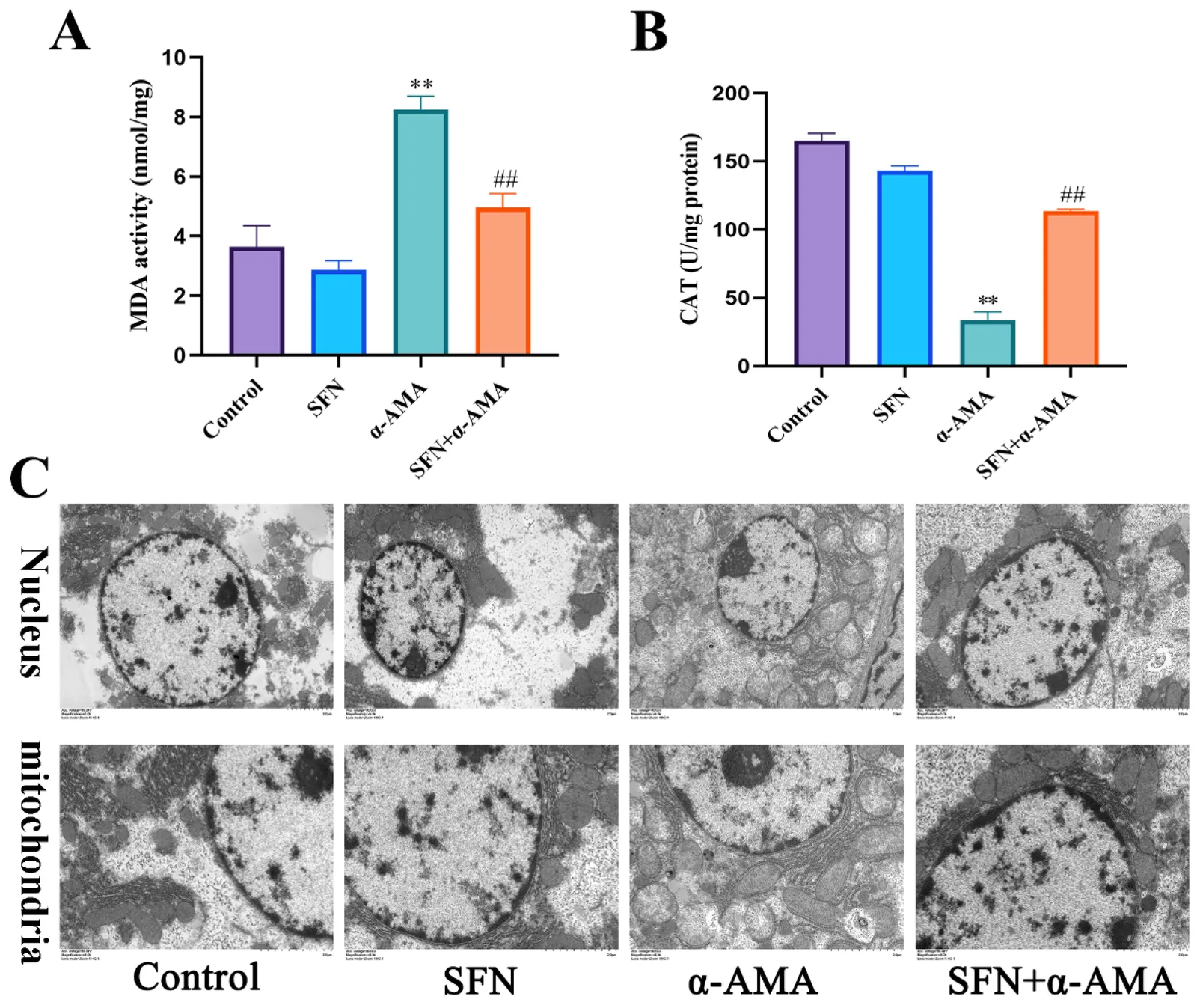
SFN alleviated α-AMA-induced oxidative stress in mouse liver. (**A**,**B**) Changes in MDA and CAT levels. (**C**) Morphological changes of nucleus and mitochondria in liver tissue (scale bar: nucleus: 5 μm, mitochondria: 2 μm). Note: *n* = 3; “*” indicates a significant difference compared with the control group (** *p* < 0.01); “#” indicates a significant difference compared with the α-AMA group (## *p* < 0.01).

To elucidate the effect of SFN on oxidative stress in mice induced by α-AMA. The MDA and CAT results were shown in Figure 2A,B. Compared with the control group, the MDA content in the α-AMA group increased significantly (*p* < 0.01); compared with the α-AMA group, the SFN+α-AMA group MDA content decreased significantly (*p* < 0.01). Compared with the control group, the CAT content in the α-AMA group decreased significantly (*p* < 0.01); compared with the α-AMA group, the SFN+α-AMA group CAT content increased significantly (*p* < 0.01). It shows that SFN alleviates α-AMA-induced oxidative stress in mouse hepatocytes on the one hand by reducing the production of MDA, and on the other hand promoting the production of CAT. The transmission electron microscopy results of liver tissue were shown in Figure 2C. Compared with the control group, the chromatin in the α-AMA group had thick edges, nuclear pyknosis, mitochondrial cristae dissolution, vacuolation, and outer membrane rupture. Compared with the α-AMA group, the chromatin in the SFN+α-AMA group was only slightly peripheralized and the mitochondria were slightly swollen.

### 3.3. The Protective Effect of SFN on Oxidative Stress and Mitochondrial Damage in AML-12 Cells Induced by α-AMA

**Figure 3:**
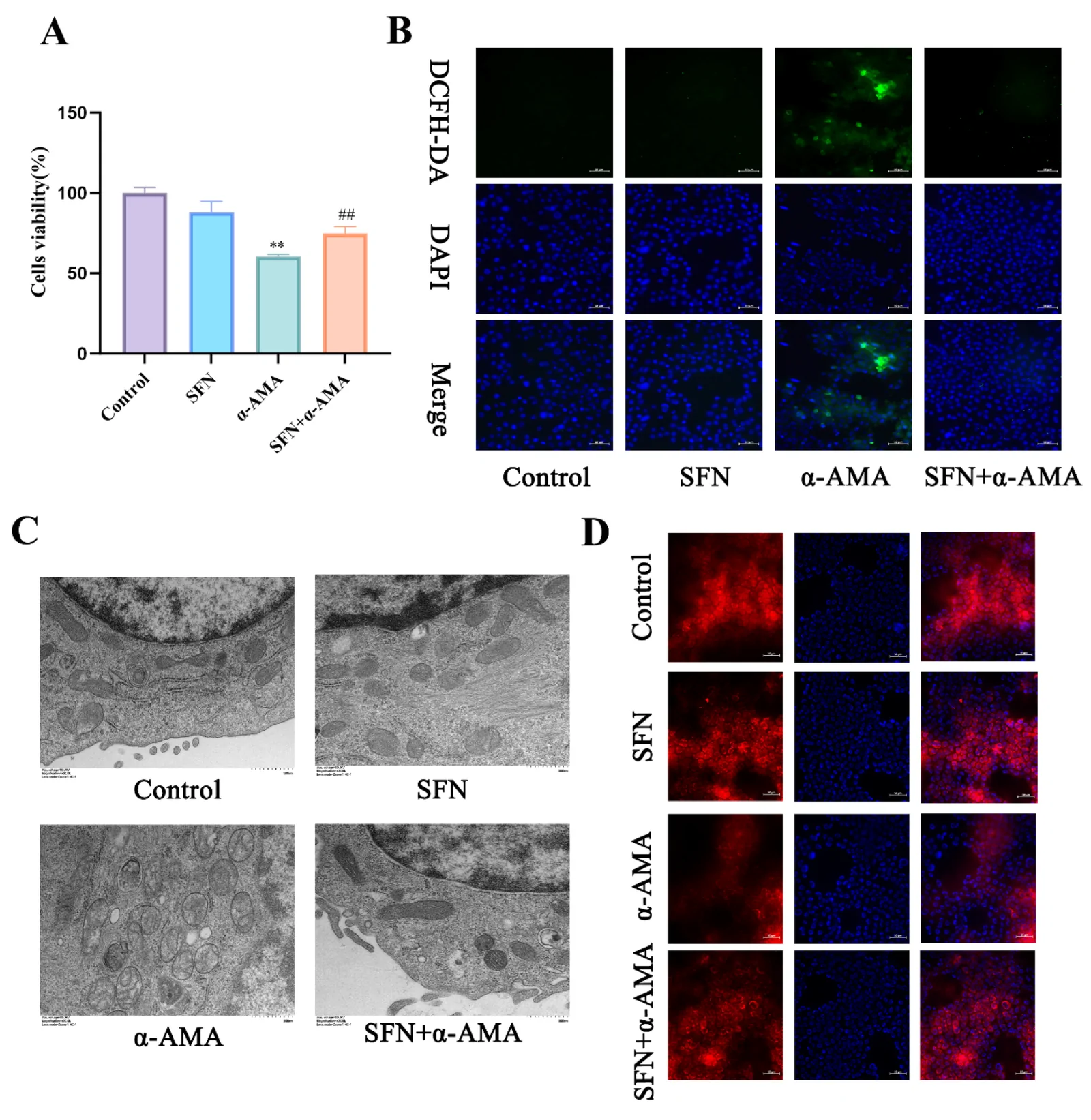
The protective effect of SFN on oxidative stress and mitochondrial damage in AML-12 cells induced by α-AMA. (**A**) CCK-8 method to detect AML-12 cell viability. (**B**) ROS levels in AML-12 cells. (scale bar: 50 μm). (**C**) Transmission electron microscope observation of morphological changes in the mitochondria of AML-12 cells (scale bar: 2 μm). (**D**) Mitochondrial membrane potential of AML-12 cells (scale bar: 50 μm). Note: *n* = 3; “*” indicates a significant difference compared with the control group (** *p* < 0.01); “#” indicates a significant difference compared with the α-AMA group (## *p* < 0.01).

The present study aims to explore the effect of SFN on α-AMA-induced AML-12 cells. The CCK-8 assay was utilised to detect alterations in the viability of AML-12 cells, with the outcomes presented in Figure 3A. In comparison with the control group, the cell viability of the α-AMA group demonstrated a significant decrease (*p* < 0.01). Conversely, the cell viability of the SFN+α-AMA group exhibited a significant increase (*p* < 0.01), suggesting that SFN can mitigate the decline in AML-12 cell viability induced by α-AMA. As demonstrated in Figure 3B, the results of the ROS level detection in AML-12 cells revealed that, in comparison with the control group, the α-AMA group exhibited bright green fluorescence, thereby indicating a significant enhancement in the fluorescence intensity of ROS in AML-12 cells. In comparison with the α-AMA group, the fluorescence intensity of ROS in AML-12 cells in the SFN+α-AMA group was significantly decreased, indicating that SFN can inhibit the generation of ROS in AML-12 cells induced by α-AMA and reduce the oxidative stress damage of AML-12 cells caused by α-AMA.

The mitochondrial alterations in AML-12 cells were examined using a transmission electron microscope, and the observations are presented in Figure 3C. The mitochondria in the control and SFN groups exhibited typical morphologies, with intact membranes, clearly visible cristae, uniform matrix density, and intact and complete nuclear membranes. A comparison of the mitochondria in the α-AMA group with those in the control group revealed the presence of several notable abnormalities. These included swelling of the mitochondria, defective mitochondrial membranes, a thin mitochondrial matrix, the presence of apoptotic bodies, and a blurred nuclear membrane. In comparison with the α-AMA group, the SFN+α-AMA group exhibited no significant alterations in mitochondrial morphology, characterised by uniform matrix density and intact nuclear membranes. The findings demonstrate that α-AMA can induce mitochondrial impairment in AML-12 cells and that SFN can mitigate the mitochondrial damage caused by α-AMA. As demonstrated in Figure 3D, the results of the mitochondrial membrane potential detection in AML-12 cells revealed a significant decrease in the fluorescence intensity of the mitochondrial membrane potential of AML-12 cells in the α-AMA group when compared with the control group. In comparison with the α-AMA group, the fluorescence intensity of the mitochondrial membrane potential of AML-12 cells in the SFN+α-AMA group increased significantly, indicating that SFN can mitigate the decrease in mitochondrial membrane potential of AML-12 cells induced by α-AMA.

### 3.4. SFN Activated the Pink1-Parkin Signaling Pathway

**Figure 4:**
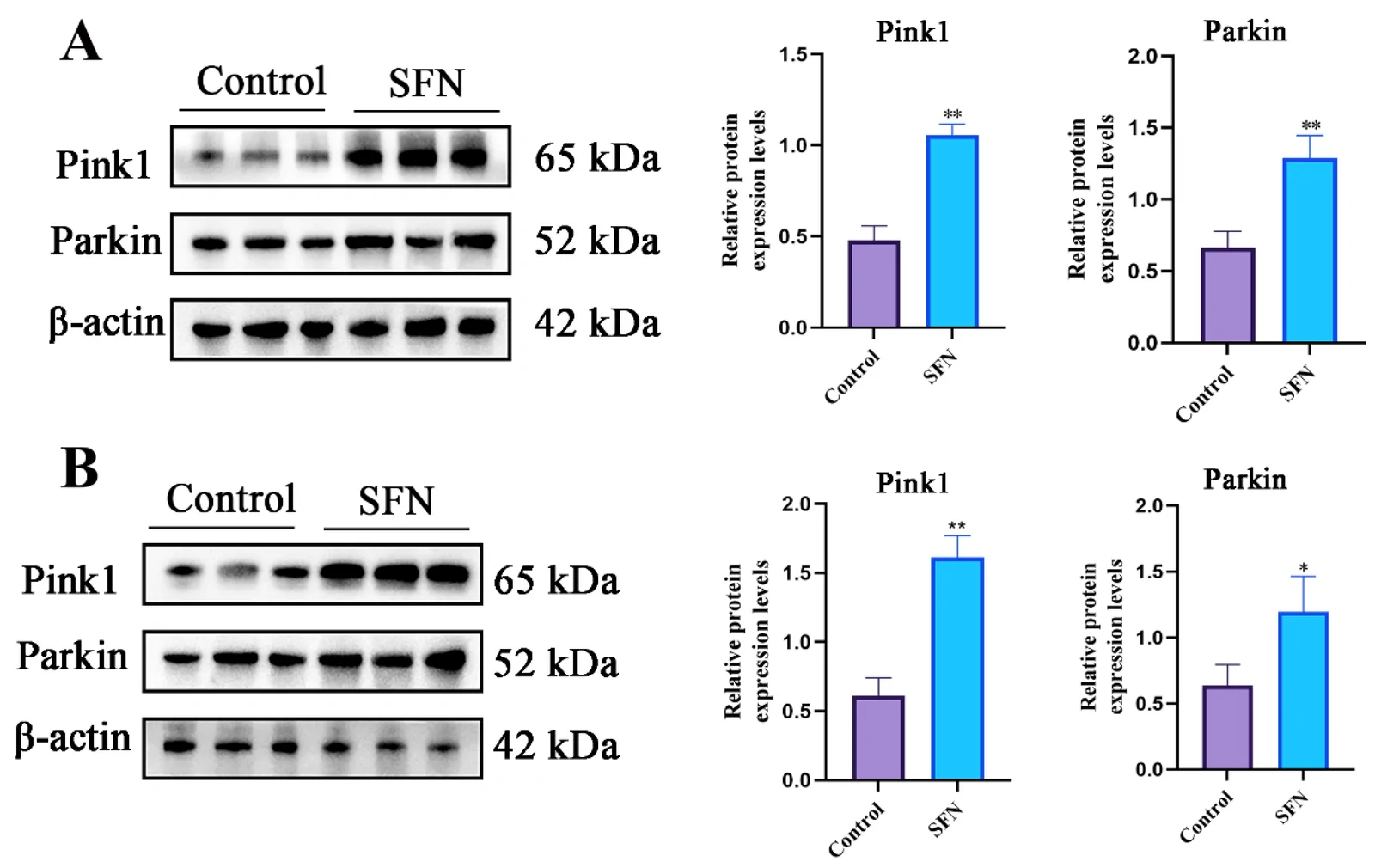
SFN increased the expression level of Pink1. (**A**) Western blot detected the expression levels of Pink1 and Parkin in mouse liver (*n* = 3). (**B**) Western blot detected the expression levels of Pink1 and Parkin in AML-12 cells (*n* = 3). Note: “*” indicates significant difference compared with the control group (* *p* < 0.05, ** *p* < 0.01).

The present study aims to explore the effect of SFN treatment on the Pink1-Parkin signalling pathway. C57BL/6J mice were administered 25 mg/kg of SFN, and one week later, Western blot analysis was employed to assess the expression levels of Pink1 and Parkin in liver tissue, as illustrated in Figure 4A. In comparison with the control group, the expression level of Pink1 in the SFN group was found to be significantly elevated (*p* < 0.01). Similarly, the expression level of Parkin in the SFN group was also found to be significantly elevated in comparison with the control group (*p* < 0.01). Western blot analysis was employed to assess the expression levels of Pink1 and Parkin in AML-12 cells, with the results depicted in Figure 4B. In comparison with the control group, the expression level of Pink1 in AML-12 cells within the SFN group exhibited a significant increase (*p* < 0.01). Similarly, the expression level of Parkin in AML-12 cells within the SFN group demonstrated a significant increase (*p* < 0.05) when compared with the control group. 

### 3.5. Pink1 Overexpression Alleviated α-AMA-Induced Oxidative Stress and Mitochondrial Damage in AML-12 Cells

**Figure 5:**
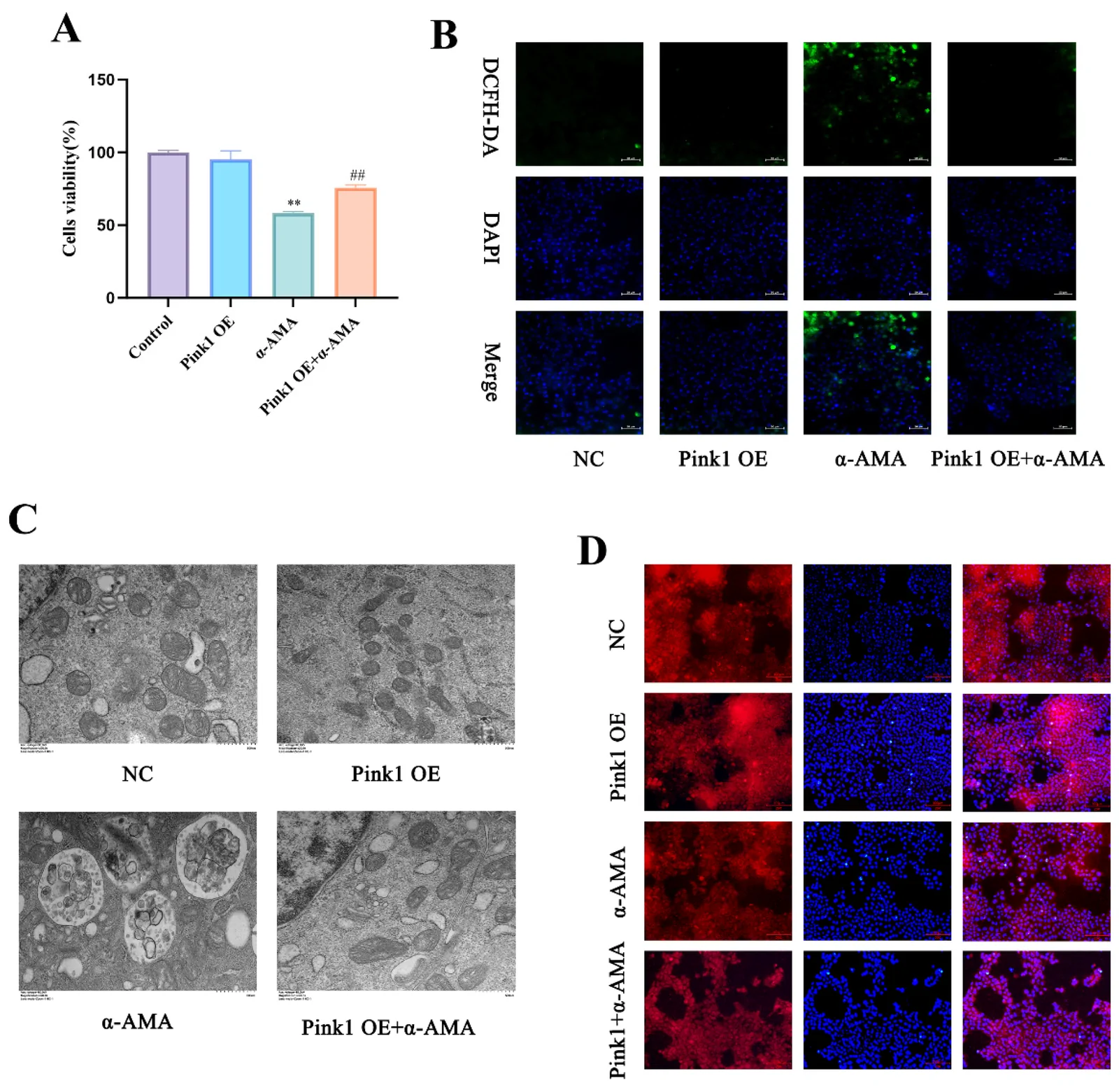
Overexpression of Pink1 alleviated α-AMA-induced oxidative stress and mitochondrial damage in AML-12 cells. (**A**) CCK-8 detected AML-12 cell viability. (**B**) ROS levels in AML-12 cells (scale bar: 50 μm). (**C**) Transmission electron microscope observation of mitochondrial morphology in AML-12 cells (scale bar: 5 μm). (**D**) Changes in mitochondrial membrane potential in AML-12 cells (scale bar = 100 μm). Note: *n* = 3; “*” indicates a significant difference compared with the control group (** *p* < 0.01); “#” indicates a significant difference compared with the α-AMA group (## *p* < 0.01).

To explore whether SFN affects α-AMA-induced oxidative stress and mitochondria in AML-12 cells by activating Pink1, a control group, Pink1 OE group, α-AMA group and Pink1 OE+α-AMA group were set up. CCK-8 detected cell viability changes, and the results were shown in Figure 5A. Compared with the control group, the cell viability of the α-AMA group decreased significantly (*p* < 0.01); compared with the α-AMA group, the cell viability of the Pink1 OE+α-AMA group increased significantly (*p* < 0.01), indicating that α-AMA inhibits AML-12 cell viability, and Pink1 overexpression can alleviate the decrease in AML-12 cell viability induced by α-AMA. Changes in intracellular ROS levels were detected, and the results were shown in Figure 5B. Compared with the control group, the α-AMA group showed bright green fluorescence, and the ROS fluorescence intensity increased significantly. Compared with the α-AMA group, the ROS fluorescence intensity of the Pink1 OE+α-AMA group decreased significantly, indicating that α-AMA induced AML-12 cells to generate excessive ROS, causing cellular oxidative stress damage. Pink1 overexpression inhibited α-AMA-induced ROS generation in AML-12 cells and alleviated oxidative stress.

The morphological changes in mitochondria in AML-12 cells were observed under transmission electron microscope, and the results were shown in Figure 5C. Most of the mitochondria in the control group and Pink1OE group were in normal shape, with clear and complete mitochondrial membranes, clearly visible mitochondrial cristae, and uniform matrix density. Compared with the control group, the mitochondria in the α-AMA group were swollen, the mitochondrial membrane was defective, the boundaries were blurred, the mitochondrial matrix was thin, the cristae were blurred and difficult to distinguish, and a large number of apoptotic bodies were seen. Compared with the α-AMA group, the mitochondrial morphology in the Pink1OE+α-AMA group changed less, the mitochondrial membrane was clearer and more complete, and the matrix density was more uniform. It shows that α-AMA can cause mitochondrial swelling in AML-12 cells and cause mitochondrial damage, while overexpression of Pink1 can reduce mitochondrial damage in AML-12 cells caused by α-AMA. The results of mitochondrial membrane potential detection were shown in Figure 5D. Compared with the control group, the fluorescence intensity of the α-AMA group was weakened, and the fluorescence intensity of the mitochondrial membrane potential was significantly weakened. Compared with α-AMA, the fluorescence intensity of the Pink1 OE+α-AMA group was enhanced, and the fluorescence intensity of the mitochondrial membrane potential was significantly enhanced, indicating that α-AMA can induce a decrease in mitochondrial membrane potential in AML-12 cells. Pink1 overexpression can alleviate the decrease in mitochondrial membrane potential in AML-12 cells caused by α-AMA.

### 3.6. SFN Alleviated α-AMA-Induced Apoptosis

**Figure 6:**
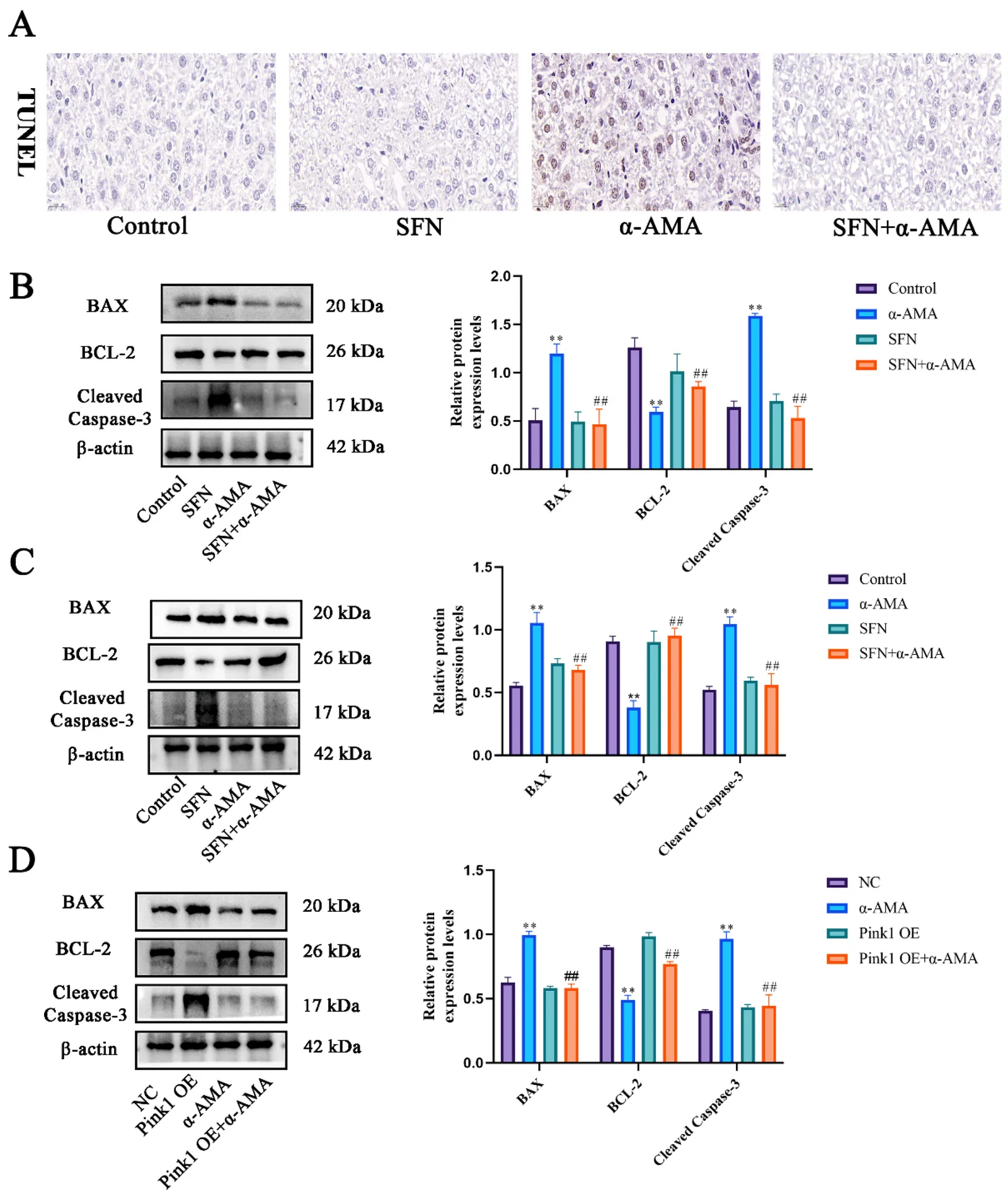
SFN alleviated α-AMA-induced apoptosis by increasing the expression level of Pink1. (**A**) TUNEL staining of mouse liver (scale bar: 50 μm). (**B**) Western blot detected the expression levels of Bax, Bcl2 and Cleaved-Caspase3 in mouse liver (*n* = 3). (**C**) Western blot detected the expression levels of Bax, Bcl2 and Cleaved-Caspase3 in AML-12 cells (*n* = 3). (**D**) Western blot detected the expression levels of Bax, Bcl2 and Cleaved-Caspase3 in AML-12 cells after Pink1 overexpression (*n* = 3). Note: “*” indicates a significant difference compared with the control group (** *p* < 0.01); “#” indicates a significant difference compared with the α-AMA group (## *p* < 0.01).

The present study aims to explore the potential of SFN in mitigating α-AMA-induced apoptosis. The results of TUNEL staining are presented in Figure 6A. A comparison of the cell nuclei in the α-AMA group with those in the control group revealed a staining pattern of brown to dark brown. In contrast, only a few cell nuclei in the SFN+α-AMA group exhibited a similar staining intensity. As demonstrated in Figure 6B, the Western blot results of liver revealed that, in comparison with the control group, the expression levels of Bax and Cleaved-Caspase3 in the α-AMA group exhibited a significant increase (*p* < 0.01). Conversely, the expression levels of Bax and Cleaved-Caspase3 in the SFN+α-AMA group demonstrated a decrease in comparison with the α-AMA group (*p* < 0.01). The results of the Western blot detection of AML-12 cells are presented in Figure 6C. In comparison with the control group, the expression levels of Bax and Cleaved-Caspase 3 in the α-AMA group were significantly increased (*p* < 0.01). Conversely, the expression levels of Bax and Cleaved-Caspase 3 in the SFN+α-AMA group were significantly decreased (*p* < 0.01).

In order to explore whether SFN can alleviate liver damage caused by α-AMA by activating Pink1, a control group, a Pink1 OE group, an α-AMA group and a Pink1 OE+α-AMA group were set up. Western blot was used to detect the expression levels of Bax, Cleaved-Caspase 3 and Bcl2 in the corresponding groups. As demonstrated in Figure 6D, the expression levels of Bax and Cleaved-Caspase3 were found to be significantly elevated in the α-AMA group in comparison to the control group (*p* < 0.01). In contrast, the expression levels of Bax and Cleaved-Caspase3 were observed to be significantly reduced in the Pink1 OE+α-AMA group when compared to the α-AMA group (*p* < 0.01).

## 4. Discussion

SFN, as a natural isothiocyanate compound, has been proven to have a wide range of protective effects in various liver injury models. In paraquat-induced acute liver injury in mice, SFN reduced mitochondrial damage and oxidative stress by reducing serum aminotransferase activity [22]; in lipopolysaccharide/D-galactosamine-induced acute liver failure model, SFN regulated HDAC6 activity to inhibit ferroptosis [23]; in the hemorrhagic shock model, SFN promoted the polarization of Kupffer cells toward the M2 phenotype, thereby regulating liver autophagy and apoptosis [24]. In addition, SFN alleviates endoplasmic reticulum stress in NAFLD [25], reverses liver ferroptosis in diabetes model [26], reduces liver injury in ischemia-reperfusion model [27], and improves liver metabolism and intestinal flora in circadian rhythm disorder model [28]. The above studies collectively indicate that SFN exerts antioxidant, anti-inflammatory and anti-apoptotic effects in various liver injury models, but none of them involve α-AMA poisoning models. In this experiment, SFN was applied to the α-AMA poisoning model, and it was found that SFN pretreatment can significantly reduce the ALT and AST levels of poisoned mice, reduce the pathological damage of liver tissue, reduce the liver MDA content and increase the CAT activity; at the cellular level, SFN also effectively improves the α-AMA-induced decrease in AML-12 cell viability, overproduction of ROS, mitochondrial swelling and decrease in membrane potential. The above results indicate that SFN has a significant protective effect on α-AMA acute liver injury.

SFN plays a protective role in various liver injury models such as paraquat-induced liver injury in mice [22], lipopolysaccharide/D-galactosamine-induced liver failure [23], NAFLD [25] and ischemia-reperfusion [27]. It mainly exerts antioxidant, anti-inflammatory and anti-apoptotic effects through Nrf2, AMPK, NF-κB and other pathways. The mechanism of liver injury induced by α-AMA is complex, and studies have found that its mechanism not only involves RNA polymerase II inhibition, but is also closely related to oxidative stress, mitochondrial damage and apoptosis [29,30]. Some studies have shown that cannabidiol can alleviate oxidative stress and apoptosis induced by α-AMA by increasing Nrf2 [31], but other studies have found that α-AMA can increase the expression of GSTA1 after activating NRF2, and instead exacerbates hepatotoxicity by consuming glutathione [32], suggesting that simply activating Nrf2 is not necessarily beneficial, and more precise intervention targets need to be found. It is worth noting that studies have confirmed that Pink1-Parkin can reduce α-AMA-induced hepatocyte damage [21].

The Pink1–Parkin axis is essential for mitochondrial quality control and cellular homeostasis, and in recent years it has been demonstrated to exert protective effects in various models of hepatic injury. In hepatic ischemia–reperfusion injury, liver regeneration enhancer factor attenuates mitochondria-derived ROS production and NLRP3 inflammasome activation via this axis [33]; FGF4 suppresses hepatocyte apoptosis by activating CaMKKβ–Pink1 signaling, thereby protecting against immune-mediated liver damage [34]; Metformin ameliorates hepatic metabolic dysfunction in mice with polycystic ovary syndrome by activating the Ethe1–Keap1–Pink1 cascade [35]. However, some studies have also found that deoxynivalenol inhibits the p62-Keap1-Nrf2 pathway by overactivating Pink1-Parkin-mediated mitophagy, which in turn aggravates liver damage [36]. The above studies all support the core regulatory position of the Pink1-Parkin pathway in liver injury, and suggest that moderate activation of this pathway is crucial. This study found that SFN can significantly increase the protein expression levels of Pink1 and Parkin in the liver and cells in the α-AMA poisoning model, and Pink1 overexpression experiments confirmed that activating Pink1 can simulate the protective effect of SFN. In this experiment, the activation of the Pink1-Parkin pathway by SFN exerted a protective effect within a moderate range, and was further manifested by reducing the expression levels of Bax and Cleaved-Caspase3. This suggests that SFN can alleviate hepatocyte damage through its regulation of the Pink1-Parkin pathway and apoptosis., which is consistent with the finding of “overactivation is harmful” in previous studies [36].

## 5. Conclusions

In summary, SFN exerts a protective effect against α-AMA-induced liver injury, which is associated with its regulation of Pink1-Parkin and apoptosis. This study provides a novel theoretical foundation for the further development of detoxification strategies for mushroom poisoning based on mitochondrial regulation.

## Data Availability

The raw data supporting the conclusions of this article will be made available by the authors on request.

## References

[1] Mao B., Ren B., Wu J., Tang X., Zhang Q., Zhao J., Zhang L., Chen W., Cui S. (2023). The Protective Effect of Broccoli Seed Extract against Lipopolysaccharide-Induced Acute Liver Injury via Gut Microbiota Modulation and Sulforaphane Production in Mice. Foods.

[2] Yang J., Guo X., Li T., Xie Y., Wang D., Yi L., Mi M. (2023). Sulforaphane Inhibits Exhaustive Exercise-Induced Liver Injury and Transcriptome-Based Mechanism Analysis. Nutrients.

[3] Mansouri R.A., Alshaibi H.F., Alqurashi M.M., Shaikh M.M., Bahaidrah K.A., Alzahrani N.A. (2024). Sulforaphane protects against LPS-induced liver injury in mice by antagonizing oxidative stress and apoptosis through AMPK activation. Int. J. Health Sci..

[4] Ma S., Pang X., Tian S., Sun J., Hu Q., Li X., Lu Y. (2022). The protective effects of sulforaphane on high-fat diet-induced metabolic associated fatty liver disease in mice *via* mediating the FXR/LXRα pathway. Food Funct..

[5] Xu Y., Huang X., Huangfu B., Hu Y., Xu J., Gao R., Huang K., He X. (2023). Sulforaphane Ameliorates Nonalcoholic Fatty Liver Disease Induced by High-Fat and High-Fructose Diet via LPS/TLR4 in the Gut-Liver Axis. Nutrients.

[6] Yang W., Zeng S., Shao R., Jin R., Huang J., Wang X., Liu E., Zhou T., Li F., Chen Y. (2025). Sulforaphane regulation autophagy-mediated pyroptosis in autoimmune hepatitis via AMPK/mTOR pathway. Int. Immunopharmacol..

[7] Xie S., Liu M., Chen L., Xie Y., Liu L., Chen W., Huang H. (2025). Sulforaphane alleviates hepatocyte pyroptosis via activating Nrf2-HO-1 signaling during septic acute liver injury. Front. Pharmacol..

[8] Wang H., Zhang X., Li X., Zhang X., Sun T., Wang Y., Zheng X., Zhao X., Liu J., Wang S. (2025). Understanding α-amanitin hepatotoxicity: Mechanisms from cellular models. Toxicology.

[9] Zhao Z., Yi S., E H., Jiang L., Zhou C., Zhao X., Yang L. (2024). α-amanitin induce inflammatory response by activating ROS/NF-κB-NLRP3 signaling pathway in human hepatoma HepG2 cells. Chemosphere.

[10] Wang H., Zhang H., Miao L., Wang C., Teng H., Li X., Zhang X., Yang G., Wang S., Zeng X. (2025). α-amanitin induces hepatotoxicity via PPAR-γ inhibition and NLRP3 inflammasome activation. Ecotoxicol. Environ. Saf..

[11] Cheng Z., Cheng K., Tang Y., Duan X., Fu Y., Duan H., Ye Y. (2025). α-Amanitin aggravates hepatic injury by activating oxidative stress and mitophagy via peroxiredoxin 6 inhibition. Immunol. Res..

[12] Lin L.Y., Tong Y.L., Lu Y.Q. (2022). The characteristics of liver injury induced by Amanita and clinical value of α-amanitin detection. Hepatobiliary Pancreat. Dis. Int..

[13] Gezer A., Ustundag H., Mendil A.S., Bedir G., Duysak L. (2024). Hepatoprotective effects of resveratrol on α-amanitin-induced liver toxicity in rats. Toxicon Off. J. Int. Soc. Toxinol..

[14] Zheng C., Lv S., Ye J., Zou L., Zhu K., Li H., Dong Y., Li L. (2023). Metabolomic Insights into the Mechanisms of Ganoderic Acid: Protection against α-Amanitin-Induced Liver Injury. Metabolites.

[15] He H., Tang Y., Zhuang L., Zheng Y., Huang X. (2024). PINK1/Park2-Mediated Mitophagy Relieve Non-Alcoholic Fatty Liver Disease. Physiol. Res..

[16] Yao Z., Li X., Wang W., Ren P., Song S., Wang H., Xie Y., Li X., Li Z. (2023). Corn peptides attenuate non-alcoholic fatty liver disease via PINK1/Parkin-mediated mitochondrial autophagy. Food Nutr. Res..

[17] Xu W., Nie L., Cao J., Shan C., Cao Y., Yu J., Tuo P., Yu J., Fujii H., Thuy L.T.T. (2026). Myricetin stabilizes PINK1 to activate mitophagy and ameliorate metabolic dysfunction-associated steatotic liver disease. Phytomed. Int. J. Phytother. Phytopharm..

[18] Li R., Wang Z., Wang Y., Sun R., Zou B., Tian X., Liu D., Zhao X., Zhou J., Zhao Y. (2023). SIRT3 regulates mitophagy in liver fibrosis through deacetylation of PINK1/NIPSNAP1. J. Cell. Physiol..

[19] Wang Q., Jia F., Guo C., Wang Y., Zhang X., Cui Y., Song M., Cao Z., Li Y. (2022). PINK1/Parkin-mediated mitophagy as a protective mechanism against AFB_1_-induced liver injury in mice. Food Chem. Toxicol..

[20] Song C., Zhang A., Zhang M., Song Y., Huangfu H., Jin S., Sun Y., Zhang C., Shi D., Wang J. (2023). Nrf2/PINK1-mediated mitophagy induction alleviates sodium fluoride-induced hepatic injury by improving mitochondrial function, oxidative stress, and inflammation. Ecotoxicol. Environ. Saf..

[21] Zhao Y., Zou Z., Wu P., Li Z., Liu Z., Yi Z., Wang J., Wu Y., Qu W., Tengjaroensakul B. (2026). PINK1-Parkin-Mediated mitophagy alleviates hepatocyte injury induced by α-amanitin. Ecotoxicol. Environ. Saf..

[22] Wang J., Peng L., Wu L., Huang S., He G., Shen P., Liang J., Huang T., Huang J., Zhong H. (2024). Sulforaphane alleviates acute liver injury induced by diquat in mice by activating Keap1/Nrf2 signaling pathway. Zhonghua Wei Zhong Bing Ji Jiu Yi Xue.

[23] Zhang Y.Q., Shi C.X., Zhang D.M., Zhang L.Y., Wang L.W., Gong Z.J. (2023). Sulforaphane, an NRF2 agonist, alleviates ferroptosis in acute liver failure by regulating HDAC6 activity. J. Integr. Med..

[24] Qin K., Liang W., Fragoulis A., Yan W., Zhang X., Zhao Q., Ma C., He Z., Buhl E.M., Li Y. (2025). Sulforaphane regulates hepatic autophagy and apoptosis by modulating Kupffer cells’ polarization via Nrf2/HO-1 pathway in the murine hemorrhagic shock/resuscitation model. Eur. J. Trauma Emerg. Surg..

[25] Mansour S.Z., Moustafa E.M., Moawed F.S.M. (2022). Modulation of endoplasmic reticulum stress via sulforaphane-mediated AMPK upregulation against nonalcoholic fatty liver disease in rats. Cell Stress Chaperones.

[26] Savic N., Markelic M., Stancic A., Velickovic K., Grigorov I., Vucetic M., Martinovic V., Gudelj A., Otasevic V. (2024). Sulforaphane prevents diabetes-induced hepatic ferroptosis by activating Nrf2 signaling axis. BioFactors.

[27] Nakatake R., Okuyama T., Hashimoto Y., Ishizaki M., Yanagida H., Kitade H., Yoshizawa K., Nishizawa M., Sekimoto M. (2024). Sulforaphane Is Protective against Warm Ischemia/Reperfusion Injury and Partial Hepatectomy in Rats. Int. J. Mol. Sci..

[28] He C., Chen M., Jiang X., Ren J., Ganapathiraju S.V., Lei P., Yang H., Pannu P.R., Zhao Y., Zhang X. (2024). Sulforaphane Improves Liver Metabolism and Gut Microbiota in Circadian Rhythm Disorder Mice Models Fed With High-Fat Diets. Mol. Nutr. Food Res..

[29] Kim D., Lee M.S., Kim N.D., Lee S., Lee H.S. (2023). Identification of α-amanitin effector proteins in hepatocytes by limited proteolysis-coupled mass spectrometry. Chem.-Biol. Interact..

[30] Lee J., Lee B.I., Choi J., Park Y., Park S.J., Park M., Lim J.H., Hwang S., Lee J.M., Shin Y.G. (2022). Investigation of In Vitro and In Vivo Metabolism of α-Amanitin in Rats Using Liquid Chromatography-Quadrupole Time-of-Flight Mass Spectrometric Method. Molecules.

[31] Wang H., Yang G., Zhang X., Zhang H., Liu Y., Wang C., Miao L., Li Y., Huang Y., Teng H. (2023). Cannabidiol protects the liver from α-Amanitin-induced apoptosis and oxidative stress through the regulation of Nrf2. Food Chem. Toxicol. Int..

[32] Liu T., Zhang X., Zhang W., Yang D., Li J., Liu L. (2026). GSTA1 depletes glutathione and exacerbates oxidative stress in α-Amanitin-induced hepatotoxicity. Chem.-Biol. Interact..

[33] Zhu D., Zhong J., Gong X., Wu X. (2023). Augmenter of liver regeneration reduces mitochondria-derived ROS and NLRP3 inflammasome activation through PINK1/Parkin-mediated mitophagy in ischemia-reperfusion-induced renal tubular injury. Apoptosis Int. J. Program. Cell Death.

[34] Huang Z., Pan T., Xu L., Shi L., Ma X., Zhou L., Wang L., Wang J., Zhu G., Chen D. (2024). FGF4 protects the liver from immune-mediated injury by activating CaMKK*β*-PINK1 signal pathway to inhibit hepatocellular apoptosis. Acta Pharm. Sin. B.

[35] Xie Y., Tian Y., Huang J., Deng W., Li X., Liu Y., Liu H., Gao L., Xie Q., Yu Q. (2025). Metformin Alleviates Liver Metabolic Dysfunction in Polycystic Ovary Syndrome by Activating the Ethe1/Keap1/PINK1 Pathway. Int. J. Biol. Sci..

[36] Yu W., Zhang B., Yang M., Li Y., Cao Z. (2025). Deoxynivalenol induces liver injury by inhibiting the p62-Keap1-Nrf2 signaling pathway via overactivation of PINK1/Parkin-mediated mitophagy. J. Hazard. Mater..

